# MicroRNA-451 and Genistein Ameliorate Nonalcoholic Steatohepatitis in Mice

**DOI:** 10.3390/ijms20236084

**Published:** 2019-12-03

**Authors:** Mailin Gan, Linyuan Shen, Yuan Fan, Ya Tan, Ting Zheng, Guoqing Tang, Lili Niu, Ye Zhao, Lei Chen, Dongmei Jiang, Xuewei Li, Shunhua Zhang, Li Zhu

**Affiliations:** 1 College of Animal Science and Technology, Sichuan Agricultural University, Chengdu 611130, China; 2 Farm Animal Genetic Resource Exploration and Innovation Key Laboratory of Sichuan Province, Sichuan Agricultural University, Chengdu 611130, China; 3 Institute of Animal Husbandry and Veterinary, Guizhou Academy of Agricultural Science, Guiyang 550005, China

**Keywords:** miR-451, genistein, NASH, hepatocarcinoma

## Abstract

Effective, targeted therapy for chronic liver disease nonalcoholic steatohepatitis (NASH) is imminent. MicroRNAs (miRNAs) are a potential therapeutic target, and natural products that regulate miRNA expression may be a safe and effective treatment strategy for liver disease. Here, we investigated the functional role of miR-451 and the therapeutic effects of genistein in the NASH mouse model. MiR-451 was downregulated in various types of liver inflammation, and subsequent experiments showed that miR-451 regulates liver inflammation via IL1β. Genistein is a phytoestrogen with anti-inflammatory and anti-oxidant effects. Interestingly, we found that the anti-inflammatory effects of genistein were related to miR-451 and was partially antagonized by the miR-451 inhibitor. MiR-451 overexpression or genistein treatment inhibited IL1β expression and inflammation. Taken together, this study shows that miR-451 has a protective effect on hepatic inflammation, and genistein can be used as a natural promoter of miR-451 to ameliorate NASH.

## 1. Introduction

The liver is the largest internal organ of mammals and one of the most active in terms of energy metabolism. It plays a role in deoxidation, glycogen storage, detoxification, and secretory protein synthesis in mammals [1,2]. The liver is also the largest digestive gland in the digestive system and is closely related to digestion and absorption [3]. Liver diseases are usually divided into viral liver disease (viral hepatitis A, B, C, D, and E, etc.) [4] and non-viral liver disease (alcoholic liver, fatty liver, drug-induced liver disease, metabolic liver disease, etc.) [5], and different liver diseases may develop into liver cancer [6,7]. On a global scale, liver disease has become an important public health issue [8,9]. Nonalcoholic fatty liver disease (NAFLD) is the most common liver disease with a global prevalence of 25% [10,11]. Nonalcoholic steatohepatitis (NASH) is considered to be the progressive form of NAFLD [12]. NAFLD/NASH is closely related to factors such as abnormal lipid metabolism, insulin resistance, oxidative stress, and genetic susceptibility [13,14]. The cause of NAFLD/NASH is highly complex and there are currently no direct treatment options [15]. Inflammation is a complication of various liver diseases, so relieving inflammation is one of the most important ways to control the development of liver disease [16,17].

MicroRNAs (miRNAs) are a class of non-coding RNAs (approximately 22 nucleotides in length) that play important roles in the regulation of target genes in many biological processes. MiR-451 functions as a tumor suppressor and many studies have shown that miR-451a exerts inhibitory effects on a variety of cancer cells [18,19]. Recent studies have shown that miR-451 is also closely related to nonalcoholic fatty liver [20] and liver cancer [21], although its mechanism of action has not been fully elucidated.

Genistein is an isoflavone phytoestrogen that is widely found in rhizomes and the seeds of legumes [22]. Genistein has many biological activities, including anti-cancer [23], anti-inflammatory [24], and anti-osteoporotic effects [25]. The regulatory networks and specific mechanisms of action of genistein are still unclear, and the underlying mechanisms by which genistein-related miRNAs alleviate inflammation have not been reported. We have previously demonstrated that genistein reverses cardiac hypertrophy in mice by modulating miR-451 expression [26]. The role of miR-451 in the anti-inflammatory effects of genistein is unknown. Therefore, we investigated the functional role of miR-451 and the therapeutic effects of genistein in NASH, both in vivo and in vitro.

## 2. Results

### 2.1. MiR-451 Is Downregulated in Liver Inflammation

**Figure 1:**
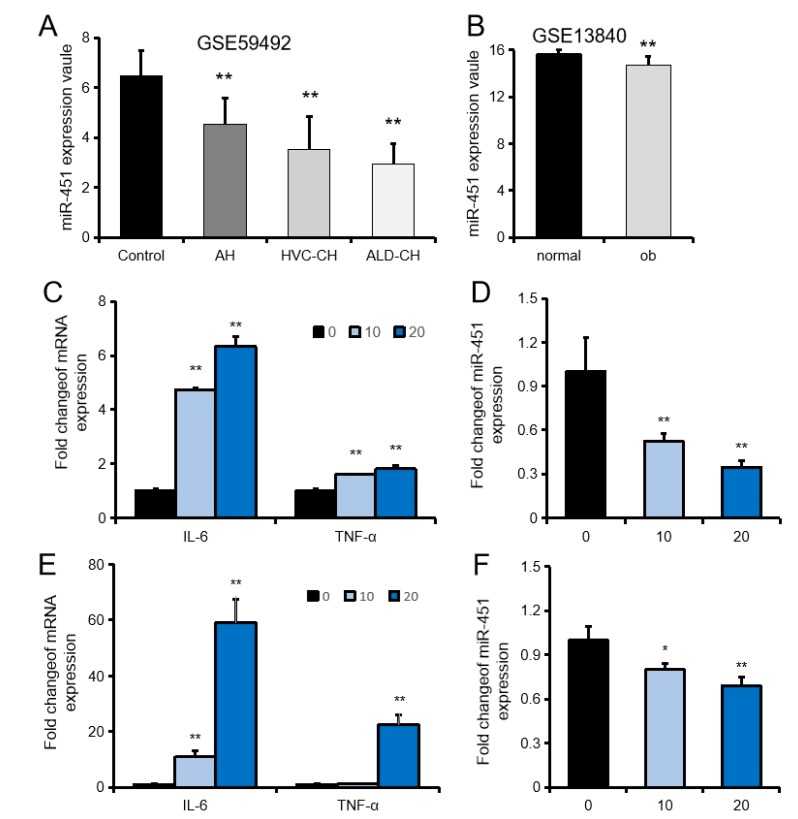
Downregulation of miR-451 in hepatic inflammation. (**A**,**B**) Expression of miR-451 in various liver diseases in the GEO database (A: GSE59492, [human]; B: GSE13840, [mouse]). Data represent means ± SEM. * *p* < 0.05, ** *p* < 0.01 compared to the control or normal groups. (**C**) The expression of IL-6 and TNF-α in NCTC1469 cells treated with LPS 10 μM and 20 μM (*n* = 3). (**D**) The expression of miR-451 in NCTC1469 cells treated with LPS 10 μM and 20 μM (*n* = 3). (**E**) The expression of IL-6 and TNF-α in Raw264.7 cells treated with LPS 10 μM and 20 μM (*n* = 3). (**F**) The expression of miR-451 in Raw264.7 cells treated with LPS 10 μM and 20 μM (*n* = 3). Data represent means ± SEM. * *p* < 0.05, ** *p* < 0.01 compared to the 0 μM LPS group.

In searching the GEO (Gene Expression Omnibus) database, we found that miR-451 was downregulated in patients with alcoholic hepatitis (AH), HCV-induced cirrhosis (HVC-CH), alcohol liver disease-induced cirrhosis (ALD-CH), and leptin-deficient obesity mice (Figure 1A,B). Furthermore, we established an in vitro liver injury model by LPS (Lipopolysaccharide) stimulation of NCTC1469 cells and Raw264.7 cells. Using this model, we showed that IL-6 and TNF-α were gradually upregulated in a dose-dependent manner, while miR-451 expression was gradually downregulated both in NCTC1469 cells (Figure 1C,D) and Raw264.7 cells (Figure 1E,F). These results indicated that miR-451 is closely related to the occurrence of liver disease.

### 2.2. MiR-451 Regulates Inflammation by Targeting IL1β

**Figure 2:**
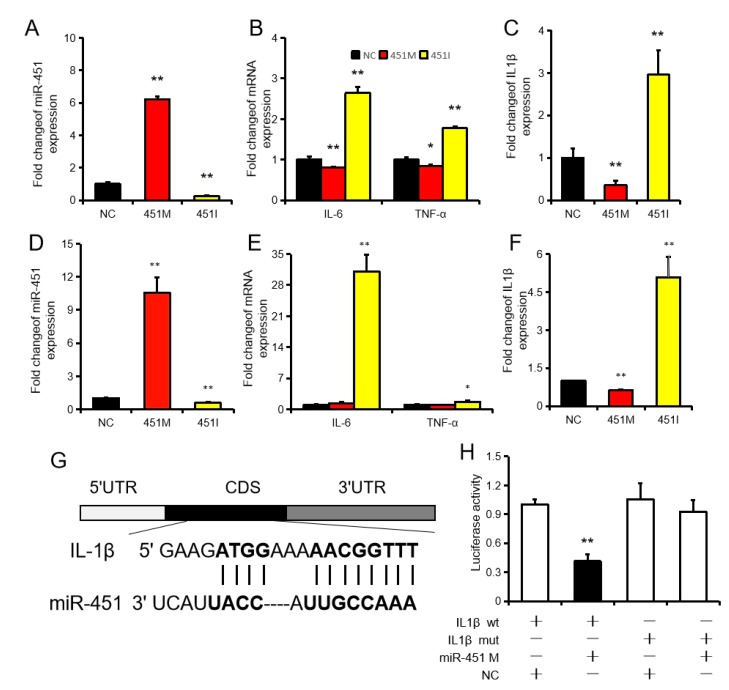
IL1β is a target gene of miR-451. (**A**) miR-451 expression in NCTC1469 cells after transfection with the miR-451 mimic, inhibitor or negative control. (**B**,**C**) The expression of IL6, TNFα, and IL1β in NCTC1469 cells after transfection with the miR-451 mimic, inhibitor, or negative control. (**D**) miR-451 expression in Raw264.7 cells after transfection with the miR-451 mimic, inhibitor or negative control. (**E**,**F**) The expression of IL6, TNFα, and IL1β in Raw264.7 cells after transfection with the miR-451 mimic, inhibitor, or negative control. (**G**) Binding site of miR-451 and IL1β. (**H**) HeLa cells were co-transfected psiCHECKTM-2 vectors and the miR-451 mimic or negative control; the luciferase activity was determined. Data represent means ± SEM. * *p* < 0.05, ** *p* < 0.01, as compared to the negative control (NC).

To further investigate the effects of miR-451 on hepatic inflammation, we successfully overexpressed miR-451 (approximately 6-fold relative to the control group) by transfection of the miR-451 mimic (Figure 2A). We found that miR-451 overexpression significantly inhibited the expression of IL6, TNFα, and IL1β in NCTC1469 cells (Figure 2B,C). However, IL6, TNFα, and IL1β expression was upregulated after inhibition of miR-451 expression in NCTC1469 cells (Figure 2B,C). We also successfully overexpressed miR-451 (approximately 10-fold relative to the control group) by transfection of the miR-451 mimic in Raw264.7 cells (Figure 2D). miR-451mimic did not affect IL6 and TNFα expression, but inhibited IL1β expression. However, transfection of the miR-451 inhibitor significantly promoted the expression of IL6, TNFα, and IL1β (Figure 2E,F).

It is worth noting that we identified a potential miR-451 binding site in the CDS (Sequence coding for aminoacids in protein) region of IL1β, which is a widely studied pro-inflammatory factor (Figure 2G). IL1β expression was significantly inhibited by the miR-451mimic and was significantly upregulated by the miR-451 inhibitor, both in NCTC1469 cells and Raw264.7 cells (Figure 2A,D). The target relationship between IL1β and miR-451 was further confirmed using dual-luciferase reporter assays. Co-transfection of HeLa cells with the wild-type luciferase plasmid and miR-451 mimics caused a significant reduction in luciferase activity compared to that in the control and mutant plasmid groups (Figure 2H).

**Figure 3:**
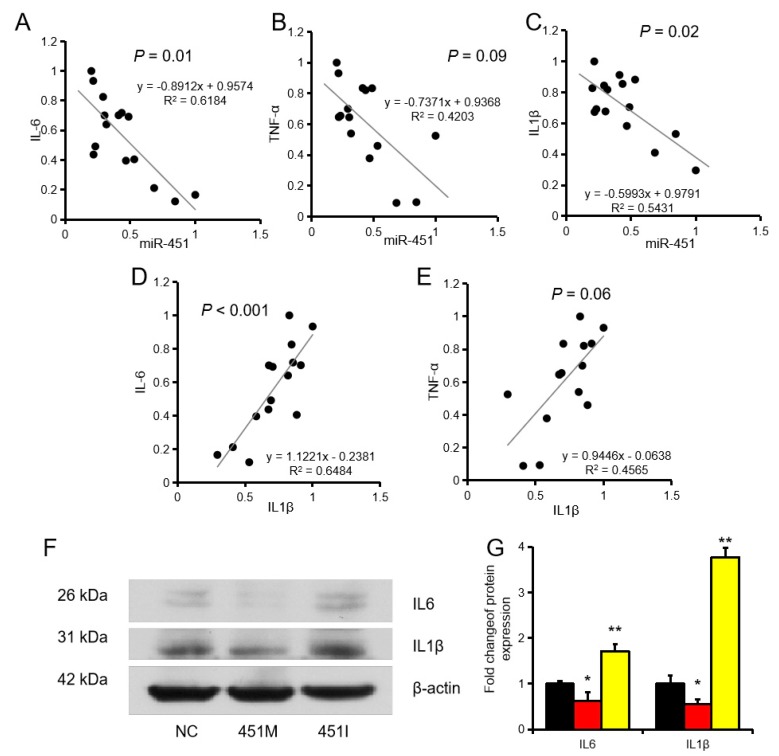
miR-451 promotes the expression of inflammatory factors. (**A**–**C**) Correlation analysis of the expression of miR-451 and IL6, TNFα, and IL1β. (**D**,**E**), (**F**,**G**) IL6 and IL1β protein levels in NCTC1469 cells. Data represent means ± SEM. * *p* < 0.05, ** *p* < 0.01, as compared to the negative control (NC).

Interestingly, correlation analysis indicated that miR-451 was significantly negatively correlated with IL6, TNFα, and IL1β levels, whereas IL1β expression showed a significantly positive correlation with IL6 and TNFα in CTC1469 cells (Figure 3A–E). In addition, we also found that mir-451mimic inhibits the protein expression of IL6 and IL1b, while miR-451inhibitor promotes the protein expression of IL6 and IL1β (Figure 3F,G). This suggested that IL1β was a direct target of miR-451. These results indicated that miR-451 plays an important role in liver inflammation.

### 2.3. Genistein Induced miR-451 Expression

**Figure 4:**
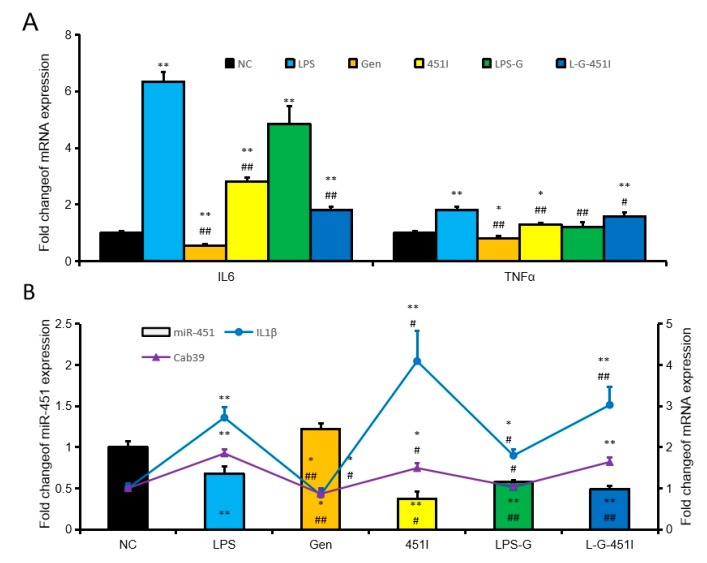
Genistein induces miR-451 to inhibit liver inflammation. (**A**) The expression of IL6 and TNFα in NCTC1469 cells after treatment with 0 μM LPS (NC), 20 μM LPS (LPS), 20 μM genistein (Gen), miR-451 inhibitor (451I), 20 μM LPS + 20 μM genistein (LPS-G), and 20 μM LPS + 20 μM genistein + miR-451 inhibitor (L-G-451I). (**B**) The expression of miR-451, IL1β, and Cab39 in NCTC1469 cells. Data represent means ± SEM. * *p* < 0.05, ** *p* < 0.01, compared to the NC group. # *p* < 0.05, ## *p* < 0.01, compared to the LPS group.

Our previous studies showed that genistein upregulates miR-451 expression in cardiomyocytes. We hypothesized that genistein also regulates the expression of miR-451 in hepatocytes. To test our hypothesis, we treated NCTC1469 cells with LPS, the miR-451 inhibitor and genistein alone or in combination. Interestingly, genistein treatment inhibited expression of IL6 and TNFα, while treatment with LPS or the miR-451 inhibitor promoted expression of IL6 and TNFα (Figure 4A). In addition, genistein antagonized the upregulation of IL6 and TNFα induced by the miR-451 inhibitor alone or the combination of the miR-451 inhibitor and LPS (Figure 4A). We also found an inverse correlation in the expression of miR-451 and its target genes IL1β and Cab39 between the different treatment groups (Figure 4B). This confirms that genistein promotes miR-451 expression in hepatocyt.

### 2.4. Genistein Inhibits LPS-Induced Liver Inflammation in Mice

**Figure 5:**
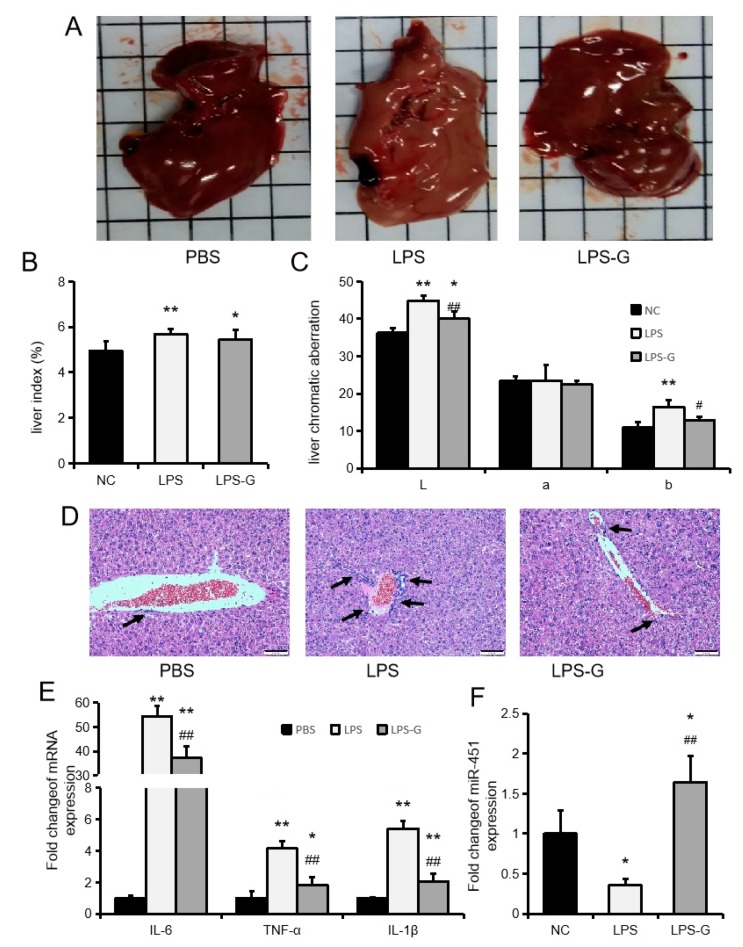
Genistein inhibits LPS-induced hepatic inflammatory responses. (**A**) Images of the whole livers of mice. (**B**) Liver weight index. (**C**) Chromatic aberration. (**D**) HE staining. The bars of the charts indicate 50 µm. (**E**) The expression of IL6, TNFα, and IL1β. (**F**) MiR-451 expression. Data represent means ± SEM. * *p* < 0.05, ** *p* < 0.01, compared to the PBS group. # *p* < 0.05, ## *p* < 0.01, compared to the LPS group.

We used LPS to construct a mouse model of acute liver inflammation to investigate the mechanism by which genistein regulates the expression of miR-451 and its effect on acute inflammation in the mouse liver. In this study, genistein attenuated the LPS-induced increases in the liver indexes L (brightness) and b (yellowness) (Figure 5A–C). HE staining of liver sections revealed a marked leukocyte infiltration of the portal area in the LPS group, whereas few infiltrating inflammatory cells were found in the LPS-G group (Figure 5D). Genistein also inhibited the LPS-induced upregulation of IL6, TNFα, and IL1β levels in the liver (Figure 5E). In addition, genistein rescued the LPS-induced downregulation of miR-451 expression (Figure 5F). These results indicated that genistein alleviates LPS-induced mice liver injury and acute inflammation.

### 2.5. Genistein Ameliorates NASH in Mice

**Figure 6:**
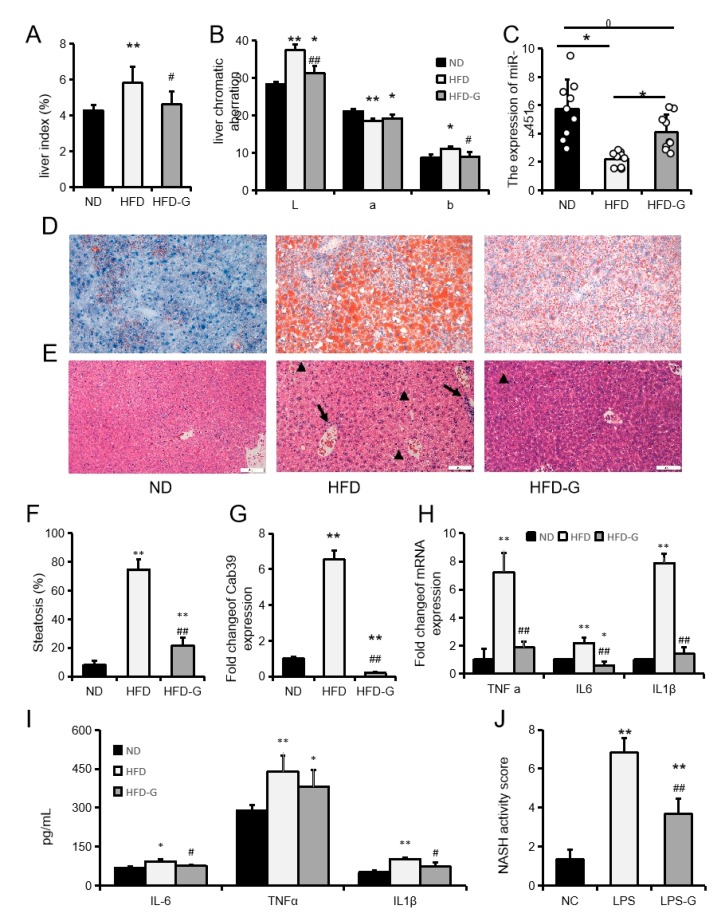
Genistein inhibits high-fat diet (HFD)-induced nonalcoholic steatohepatitis (NASH). (**A**) Liver weight index. (**B**) Chromatic aberration. (**C**) The expression of miR-451 in mice liver after treated with HFD and genistein. (**D**,**E**) The mice liver of oil red O (D) and HE (E) staining. The bars of the charts indicate 50 µm. (**F**) Steatosis degree of mice liver in each group. ((**G**,**H**) The expression of Cab39 (**G**), TNFα, IL6, and IL1β (**H**) in mice liver tissues were detected by RT-qPCR. (**I**) Liver homogenate concentrations of TNFα, IL6, and IL1β. Data represent means ± SEM. (**J**) NASH activity score of mice in each group. * *p* < 0.05, ** *p* < 0.01, compared to the ND group. # *p* < 0.05, ## *p* < 0.01, compared to the HFD group.

A high-fat diet (HFD) significantly increased liver weight, and the L and liver indexes, while reducing the b value (redness). However, genistein effectively attenuated the effects of a HFD on liver index and liver color in mice (Figure 6A,B). In addition, genistein also rescued the HFD-induced downregulation of miR-451 expression (Figure 6C). Oil red O and HE staining of liver sections revealed severe fatty infiltration in the HFD group and a significant reduction in fat infiltration in the HFD-G group (Figure 6D–F). A HFD significantly inhibited miR-451 expression and promoted the expression of Cab39, which is another miR-451 target gene. Genistein restored miR-451 expression levels and significantly inhibited Cab39 expression (Figure 6G). The expression of IL6, TNFα, and IL1β in the liver was also consistent with liver fat infiltration (Figure 6H,I). Genistein also reduces the HFD-induced NASH activity score rise (Figure 6J). These results indicated that genistein effectively inhibits HFD-induced NASH inflammatory responses and liver lipid accumulation.

### 2.6. The Potential Role of Genistein, miR-451 and Its Target Genes in the Treatment of Liver Cancer

**Figure 7:**
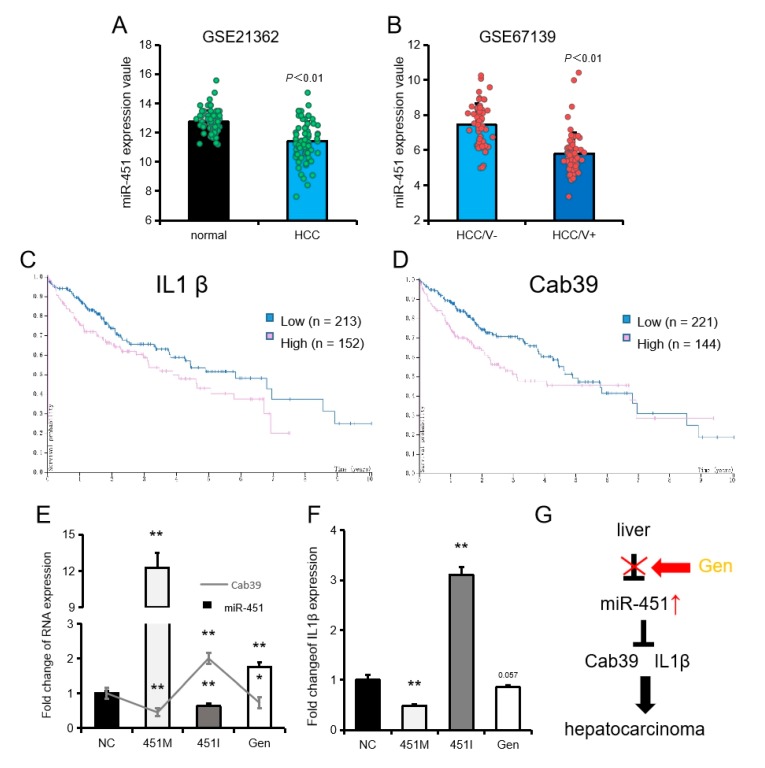
Effect of miR-451 and its target genes on liver cancer. (**A**) miR-451 expression in healthy individuals and HCC (hepatocellular carcinoma) patients in the GEO database (GSE21362). (**B**) miR-451 in HCC patients with and without vascular invasion (HCC/V+ and HCC/V-, respectively) in the GEO database (GSE67139). (**C**,**D**) The overall survival rate of patients with liver cancer expressing high and low levels of IL1β and Cab39 identified in the Human Protein Atlas database. (**E**,**F**) The expression of miR-451, Cab39, and IL1β in HePG2 cells after treatment with miR-451 mimic, miR-451 inhibitor, and genistein. Data represent means ± SEM. * *p* < 0.05, ** *p* < 0.01, compared to the NC group. (**G**) Schematic diagram showing the theoretical mechanism by which genistein functions as a potential anti-hepatocarcinoma drug.

In the GEO database, we also found that miR-451 was significantly downregulated in patients with liver cancer and further downregulated in patients with liver cancer and vascular invasion (Figure 7A,B). By screening the Human Protein Atlas database, we also found that liver cancer patients with lower expression levels of IL1β and Cab39 had higher survival rates (Figure 7C,D). In HePG2, we also found that miR-451 expression was negatively associated with the expression of its target genes IL1β and Cab39 (Figure 7E,F). These results suggested that miR-451 is closely related to the occurrence and development of liver cancer. Genistein may be a natural miR-451 promoter with potential value in the treatment of liver cancer (Figure 7G).

## 3. Discussion

MiR-451 is closely related to the occurrence and prognosis of various cancers [27] and is also associated with autoimmune inflammation [28]. In recent years, it has been reported that miR-451 is involved in the development of NAFLD [29]. However, the role of miR-451 in NAFLD/NASH has not been fully resolved, and further studies are required to elucidate its mechanism. Interestingly, searches of the GEO database revealed that miR-451 expression is downregulated in various liver diseases.

Inflammation is a common feature of disease and an important indicator of prognosis and treatment. The LPS that constitutes the cell wall of gram-negative bacilli can stimulate a strong inflammatory response and is also an important factor in liver damage [30]. A large number of studies have shown that LPS induces oxidative stress [31], participates in insulin resistance [32], and liver inflammation damage [33], and plays a key role in the pathogenesis of NAFLD/NASH [34,35]. In this study, we constructed a cell injury model by stimulation of NCTC1469 cells with LPS. Using this model, we found that TNFα and IL6 increased with LPS concentration in a dose-dependent manner, while miR-451 decreased. These findings suggest that miR-451 plays an important role in liver disease.

MicroRNAs (miRNAs) are small, non-coding RNAs that perform biological functions primarily by inhibiting target gene expression [36], mainly by combining with the 3′-UTR regions [37]. In recent years, studies have shown that miRNAs also inhibit target gene expression by binding to the target gene CDS region [38] or even the 5′-UTR region [39]. In this study, we demonstrate that miR-451 binds to the CDS region of IL1β and inhibits its expression. IL1β is a widely studied pro-inflammatory cytokine [40], and a large number of studies have confirmed that IL1β is involved in the development of NAFLD/NASH [41,42]. Cab39 is a component of the active LKB1/STRAD/Cab39 complex involved in the expression of inflammatory factors and has been identified as a target gene of miR-451 [43]. In the present study, inhibition of miR-451 expression and LPS treatment resulted in increased IL1β and Cab39 levels, while miR-451 overexpression and genistein treatment inhibited the expression of IL1β and Cab39. Furthermore, the pattern of IL1β and Cab39 expression showed a negative correlation with miR-451 levels between different treatments. This pattern of expression further confirmed the relationship between miR-451 and its target genes. Our results provide direct evidence of the inhibitory effect of miR-451 on liver inflammation.

Genistein has been reported to have broad anti-inflammatory effects [44]. Previous studies have shown that genistein improves liver diseases, such as liver hypertrophy [45], fat infiltration [46], and increased inflammation [47]. Interestingly, miR-451 is a blood-specific microRNA [48] that has also been reported as a marker of environmental hormones, such as genistein, in mammals [49,50]. We have previously demonstrated that genistein modulates the expression of mouse heart miR-451 to protect against cardiac hypertrophy [26]. In addition, miR-451 showed the most marked changes in expression, and these changes were most consistent with the changes in inflammation and lipid deposition in the three experimental groups. MiR-451 expression was significantly increased after genistein treatment both in vivo and in vitro. In the NASH model, the color of the liver changed dramatically. Color changes, which are an important indicator of the normality of tissues and organs [51] are widely quantified in food and medical fields by colorimetry [52]. Here, evaluation of liver color yellowing and tissue inflammatory infiltration showed that genistein exerted a protective effect on LPS-induced acute liver injury and HFD-induced NASH that was similar to that of miR-451 ago-miRNA [29]. These results suggest that miR-451 is involved in the mechanism by which genistein protects against NASH. It is worth noting that genistein exerted significantly protective effects and significant changes in miR-451 expression levels in both the acute and chronic inflammation models. Furthermore, our observations suggest that miR-451 not only mediates a rapid response to acute hepatic inflammation, but also a sustained effect on chronic hepatic inflammation. Thus, the use of miR-451 as a marker for liver disease has great potential.

Chronic hepatitis often develops into liver cirrhosis and increases the risk of hepatocellular carcinoma [53]. Genistein has also been reported to have an inhibitory effect on cancer [54]. In liver cancer patients, miR-451 expression was significantly downregulated, and miR-451 expression was further downregulated in patients with liver cancer vascular invasion [55]. IL1β has also been reported to promote the migration of liver cancer cells [56]. In the liver cancer cell line (HepG2), genistein significantly promoted miR-451 expression, and both the miR-451 mimic and genistein significantly inhibited the expression of IL1β and Cab39. In addition, the Human Protein Atlas database showed that liver cancer patients with high expressions of IL1β and Cab39 had lower survival rates. Based on the results of our study, and in view of the ability of genistein to regulate the expression of miR-451, IL1β, and Cab39, we speculate that miR-451 and its target genes are also involved in the anti-cancer effects of genistein.

## 4. Materials and Methods

### 4.1. Animals and Treatment

All the animal care was approved by the Institutional Animal Care and Use Committee of the College of Animal Science and Technology of Sichuan Agricultural University, Sichuan, China, under the permit of No. DKY-B20131403 (Ministry of Science and Technology, China, approved on 15 June 2004). Female ICR mice (aged 8 weeks) were purchased from the Chengdu Dashuo Experimental Animal Co, Ltd. (Chengdu, China). Mice were housed in standard plastic cages under controlled temperature conditions (22 °C ± 3 °C) and a natural light cycle. Animals were given free access to food and water. A stock solution of lipopolysaccharide (LPS, Sigma, St. Louis, MO, USA) was prepared in PBS at 2 g/L and stored at −20 °C. Prior to use, the stock solution was slowly thawed at room temperature and diluted to the required concentration. Genistein (Purity ≥98%, Jingzhu Biotechnology, Nanjing, China).

### 4.2. Animal Model

A model of liver injury was induced by intraperitoneal injection of LPS (10 mg/kg body weight) and the control group received the same amount of phosphate buffer solution (PBS) [57]. The LPS-G group received genistein for 14 days before LPS administration. The tissue samples were obtained 6 h after LPS treatment.

A model of nonalcoholic steatohepatitis (NASH) was induced in mice by being fed a high-fat diet (HFD; 40% fat) for 2 months. During the experimental period, mice were fed a maintenance diet (ND), a HFD, or a HFD supplemented with genistein (HFD-G, genistein: 100 mg/kg) for 1 month.

### 4.3. Tissue Section, Liver Weight Index and NASH Activity Score

For hematoxylin and eosin (HE) staining, mouse liver tissue was fixed with 4% paraformaldehyde. The tissues were then dehydrated using ethanol, and infiltrated and embedded using paraffin. For oil red O staining, sections of frozen mouse liver tissue were prepared and stained with oil red O for 20 min, and finally blocked for observation. Liver weight index = (liver weight/body weight × 100). The NASH activity score was calculated with reference to previous studies [45].

### 4.4. Colorimetric Evaluation of Liver Tissue

Changes in the color of the liver were measured using a colorimeter (CR-300, Minolta, Japan). L (0 is black and 100 is white); a (positive and negative values are shown in red and green, respectively); b (positive and negative values are shown in yellow and blue, respectively). Specifically, the liver of the mouse was removed and placed in a petri dish, and the left lobe of the liver was pointed at the light-collecting hole of the colorimeter for measurement.

### 4.5. Liver Homogenate Inflammatory Factors

The tumor necrosis factor α (TNFα), interleukin 6 (IL6), and IL1β levels were determined in liver homogenates using commercial kits (Shanghai Enzyme-linked Biotechnology Co., Ltd., Shanghai, China).

### 4.6. Cell Culture and Treatment

NCTC 1469 cells, Raw264.7, HeLa cells, and HePG2 cells (Stem Cell Bank, Chinese Academy of Science, Beijing, China) were cultured at 37 °C under 5% CO_2_. Genistein was dissolved in dimethyl sulfoxide (DMSO) at a concentration of 1 M and stored at −20 °C [58]. For use in experiments, NCTC1469 cells and HePG2 cells were stimulated with LPS (10 or 20 μM), genistein (20 μM), and transfected with miR-451 mimic, inhibitor, and negative control (Ribobio, Guangzhou, China) using LipofectamineTM 3000 (Invitrogen, Guangzhou, China).

### 4.7. Luciferase Reporter Assay

Dual luciferase reporter constructs were generated according to a previous report [59]. The IL1β sequence containing the miR-451 binding site was inserted into the psiCHECKTM-2 vector, and then the psiCHECKTM-2 vector and miR-451 mimic were co-transfected into HeLa cells using Lipofectamine TM 3000 reagent (Invitrogen). Finally, firefly fluorescence and renilla fluorescence were measured using Dual-Glo Luciferase Assay System (Promega, Madison, WI, USA).

### 4.8. Quantitative Real-Time PCR

Total RNA was extracted from mouse liver tissue and cell lines with TRIzol reagent (TaKaRa, Dalian, China) according to the manufacturer’s instructions. Quantitative real-time PCR (qRT-PCR) was performed using the SYBR Premix Ex Taq kit (TaKaRa, Dalian, China) on a CFX96 Real-Time PCR detection system (Bio-Rad, Richmond, CA, USA). Relative expression levels of mRNAs and miRNAs were calculated using the 2^−ΔΔCt^ method [60]. Expression levels of U6 and β-actin were used as the endogenous controls to normalize the expression of miRNA and mRNA, respectively. The primer sequences for qRT-PCR are listed in Supplementary Table S1.

### 4.9. Western Blot Analysis

Western blot analysis was performed using standard procedures, as described previously [61]. To obtain proteins, cells were lysed by 100 mg/mL RIPA lysis buffer containing 1% phenylmethylsulfonyl fluoride (Beyotime, Shanghai, China) on ice. Then, cell lysates were boiled in 5× SDS buffer for 5 min before being separated by 10% SDS-polyacrylamide gel, and transferred to a PVDF membrane (Bio-rad, Hercules, CA, USA). Subsequently, the membranes were blocked by TBST containing 5% non-fat dried milk for 2 h at 37 °C to avoid non-specific binding. After that, the membranes were incubated with primary antibodies overnight at 4 °C. Additionally, the immunoblot membranes were incubated with the secondary antibodies for 2 h at 37 °C after washing 3 times. The blots were visualized by DAB reagent (Boster, Wuhan, China) according to the manufacturer’s instructions. The antibodies included anti-β-actin (GB12001, Servicebio, Wuhan, China, dilution: 1:1000); anti-IL6 (GB11117, Servicebio, China, dilution: 1:1000); anti- IL 1 β (abs115412, Absin, Shanghai, China, dilution: 1:1000).

### 4.10. Statistical Analyses

All images were processed using Image-Pro Plus 6.0 software (National Institutes of Health, Bethesda, MD, USA). All quantitative results were presented as mean ± SEM. Statistical analyses were conducted using SPSS 20.0 software (IBM, Almond, NY, USA). Data were analyzed by one-way analysis of variance (ANOVA) model. The differences between the groups were analyzed using Student’s *t*-test and multiple comparison tests. The differences between the means were considered statistically significant when *p* < 0.05.

## 5. Conclusions

In summary, our study showed that miR-451 is downregulated in a variety of hepatic inflammatory diseases, and genistein ameliorates liver inflammation by increasing miR-451 levels. MiR-451 and its target genes, IL1β and Cab39, may play an important role in the mechanism by which genistein inhibits NAFLD/NASH and liver cancer. Our findings provide new insights into the mechanisms underlying the anti-NAFLD/NASH and anti-cancer effects of genistein in the liver, providing a reference for the use of genistein and miR-451 in liver diseases.

## Supplementary Materials

The following are available online at https://www.mdpi.com/1422-0067/20/23/6084/s1, Table S1: Sequences of PCR primers used in this study.
